# Validation of the Auditory Stroop Task to increase cognitive load in walking tasks in healthy elderly and persons with Parkinson’s disease

**DOI:** 10.1371/journal.pone.0220735

**Published:** 2019-08-06

**Authors:** S. Janssen, J. J. A. Heijs, W. van der Meijs, J. Nonnekes, M. Bittner, L. D. A. Dorresteijn, B. R. Bloem, R. J. A. van Wezel, T. Heida

**Affiliations:** 1 Biomedical Signals and Systems Group, MIRA Institute for Biomedical Technology and Technical Medicine, University of Twente, Enschede, The Netherlands; 2 Department of Neurology, Donders Institute for Brain, Cognition and Behaviour, Radboud University Medical Centre, Nijmegen, The Netherlands; 3 Department of Biophysics, Donders Institute of Brain, Cognition and Behavior, Radboud University, Nijmegen, The Netherlands; 4 Department of Rehabilitation, Donders Institute for Brain, Cognition and Behaviour, Radboud University Medical Centre, Nijmegen, The Netherlands; 5 Department of Neurology, Medisch Spectrum Twente, Enschede, The Netherlands; Karolinska Institutet, SWEDEN

## Abstract

**Background:**

The development of treatments for freezing of gait (FOG) in Parkinson’s disease (PD) requires experimental study set-ups in which FOG is likely to occur, and is amenable to therapeutic interventions. We explore whether the ‘Auditory Stroop Task’ (AST) can be used to increase cognitive load (and thereby elicit FOG), simultaneously with visual cues (as a therapeutic intervention for FOG). We additionally examined how these two contrasting effects might interact in affecting gait and FOG parameters.

**Objectives:**

We investigated whether: (1) the ‘Auditory Stroop Task’ (AST) influences gait in healthy elderly and persons with PD who experience FOG, and increases the frequency of FOG events among PD patients; (2) the AST and visual cues interact; and (3) different versions of the AST exert different cognitive loads.

**Methods:**

In ‘Experiment 1’, 19 healthy elderly subjects performed a walking task while performing a high and low load version of the AST. Walking with a random numbers task, and walking without cognitive load served as control conditions. In ‘Experiment 2’, 20 PD patients with FOG and 18 healthy controls performed a walking task with the AST, and no additional cognitive load as control condition. Both experiments were performed with and without visual cues. Velocity, cadence, stride length, and stride time were measured in all subjects. FOG severity was measured in patients.

**Results:**

Compared to the control conditions, the AST negatively affected all gait parameters in both patients and controls. The AST did not increase the occurrence of FOG in patients. Visual cues reduced the decline in stride length induced by cognitive load in both groups. Both versions of the AST exerted similar effects on gait parameters in controls.

**Conclusions:**

The AST is well-suited to simulate the effects of cognitive load on gait parameters, but not FOG severity, in gait experiments in persons with PD and FOG.

## Introduction

Cognitive dual tasks negatively affect gait in elderly people [1–3]. The relationship between dual tasks and gait is influenced by factors such as age, attentional resources, neurological comorbidity, and the type and complexity of the dual task applied [1, 2, 4]. Compared to age-matched healthy controls, persons with Parkinson’s disease (PD) are more susceptible to gait interference by dual tasks [5, 6]. This effect is even more pronounced in the presence of freezing of gait (FOG) [7, 8], a debilitating motor symptom occurring predominantly in advanced stages of PD [5, 9]. External cues such as transverse bars on the floor can oppose the effects of dual tasks on gait parameters in persons with PD [10].

In experimental settings, cognitive dual tasks can be applied to simulate the domestic situation where dual tasks (e.g. talking while walking) can worsen gait, or to provoke FOG in persons with PD. Meanwhile, external cues are being investigated for their beneficial effects on gait and FOG, and their usability in daily life[11–13]. In PD, dual tasks and external cues are often studied simultaneously [3, 10, 14–19]. In such studies, the cognitive task applied would ideally meet the following criteria. First, the task calls upon executive function, as this is an important determinant of FOG severity [20] and gait performance under dual task conditions [21]. Second, the task should not introduce a rhythm, to prevent interference with the external cues under investigation. Third, the paradigm does not interfere with vision in studies involving visual cues. Fourth, considering the age range in which PD occurs, the task should be insusceptible to age-related sensorineural hearing loss [22]. Fifth, the level of difficulty of the task should be independent of the level of education. Sixth, the task should provide the possibility to incorporate additional instructions (such as ‘start walking’) without having to add another task. Lastly, any interactions between the external cues and gait parameters are known. Current paradigms used to increase cognitive load [4] do not meet all of these criteria. For example, the random numbers task (RNT) applied previously [10] does not allow for additional commands to be enclosed within the task. The classic auditory Stroop task [23] involving high and low pitched sounds depends on hearing sensitivity [22]. A variant of the auditory Stroop task (AST), in which the words ‘man’ or ‘woman’ are spoken by a male or female speaker [24], is likely to be less susceptible to hearing quality and potentially fits the criteria described above.

The AST requires validation for it to be used in gait experiments in healthy elderly and PD patients.

The primary aim of this study was to assess whether the AST was effective in influencing gait parameters in healthy elderly and PD patients, and whether it would enhance the likelihood of FOG occurrence in persons with PD. The secondary aim was to assess whether visual cues interfered with the influence of the AST on gait parameters in both PD patients and controls. The tertiary aim was to assess whether the size of the cognitive load exerted by the AST could be manipulated by different versions of the task. These aims were investigated in two gait experiments. To minimize the number of conditions and trials, the AST was first validated against an established cognitive load task (random numbers task, RNT) and no additional cognitive load in healthy elderly (experiment 1). Then, the AST was compared to a control condition without cognitive load in PD patients and in controls (experiment 2). Both experiments measured the influence of the different cognitive loads on gait parameters in both the presence and absence of visual cues. Experiment 2 additionally measured FOG in PD patients.

We hypothesized that: 1) the AST would be at least as effective as the RNT in influencing gait parameters in controls; 2) the AST would influence gait in both patients and controls, and in patients the most; 3) the AST would increase FOG occurrence in patients; 4) visual cues would reduce the influence of the AST on gait both in patients and controls; and 5) a high load version of the AST would exert a larger effect on gait parameters in controls than its low load counterpart.

## Materials and methods

This study was performed in accordance with the guidelines of the Declaration of Helsinki (1964). All subjects provided written informed consent prior to inclusion. Both experiments were approved by the local ethics committee of the University of Twente. Experiment 2 was approved by the medical ethics committee Twente (NL60687.044.17) and registered in the Dutch trial registry (NTR6409).

### Experiment 1

#### Study population

20 healthy subjects (‘controls’) were included (Table 1). Inclusion criteria were: age 50 years and older, capable of walking unaided, no comorbidities affecting gait impairment, and intact vision and hearing.

**Table 1:** Clinical characteristics of the participants.

	Experiment 1	Experiment 2	
	Healthy controls	Healthy controls	PD patients
	Median (Q1 –Q3)	Median (Q1 –Q3)	Median (Q1 –Q3)
Number of participants	19*	18	20
Age (years)	65 (59.8–68.8) ^a^^,^	67.5 (62–70) ^a^^,^^b^	70.5 (63.5–73) ^b^
Gender (% male)	63.2 ^c^	50 ^c^^,^^d^	85^**d**^
Disease duration (years)			11 (7.5–16)
Years since FOG (years)			4 (2.5–6.5)
LED (mg/day)			1128 (901.5–1359)
UPDRS-part III			39.5 (31.5–47.5)
UPDRS-PIGD			4.5 (3–7)
Hoehn and Yahr (II / III)			12 / 8
MMSE			29 (27–30)
N-FOGQ			21 (16–25)
FAB			16 (15–17)

The median and first (Q1) and third (Q3) quartiles (i.e. the boundaries of the interquartile range) are given, unless stated otherwise.

Comparisons for age:

^a^ controls in experiment 1 vs. experiment 2, p>0.05

^b^ controls vs. patients in experiment 2, p>0.05. Comparisons for gender:

^c^ controls in experiment 1 vs. experiment 2, p>0.05

^**d**^ controls vs. patients in experiment 2, p = 0.035.

*Data of one participant in experiment 1 were discarded from analysis because of technical issues.

PD, Parkinson’s disease; FOG, Freezing of Gait; LED, levodopa equivalent dose; UPDRS-part III, Unified Parkinson’s Disease Rating Scale part III; UPDRS-PIGD, Unified Parkinson’s Disease Rating Scale—Postural instability and gait disorder; MMSE, mini-mental state examination (range 0–30); N-FOGQ, New Freezing of Gait Questionnaire (range 0–28); FAB, Frontal Assessment Battery (range 0–18). All questionnaires were rated while participants were OFF medication.

#### Experimental procedure

**Fig 1:**
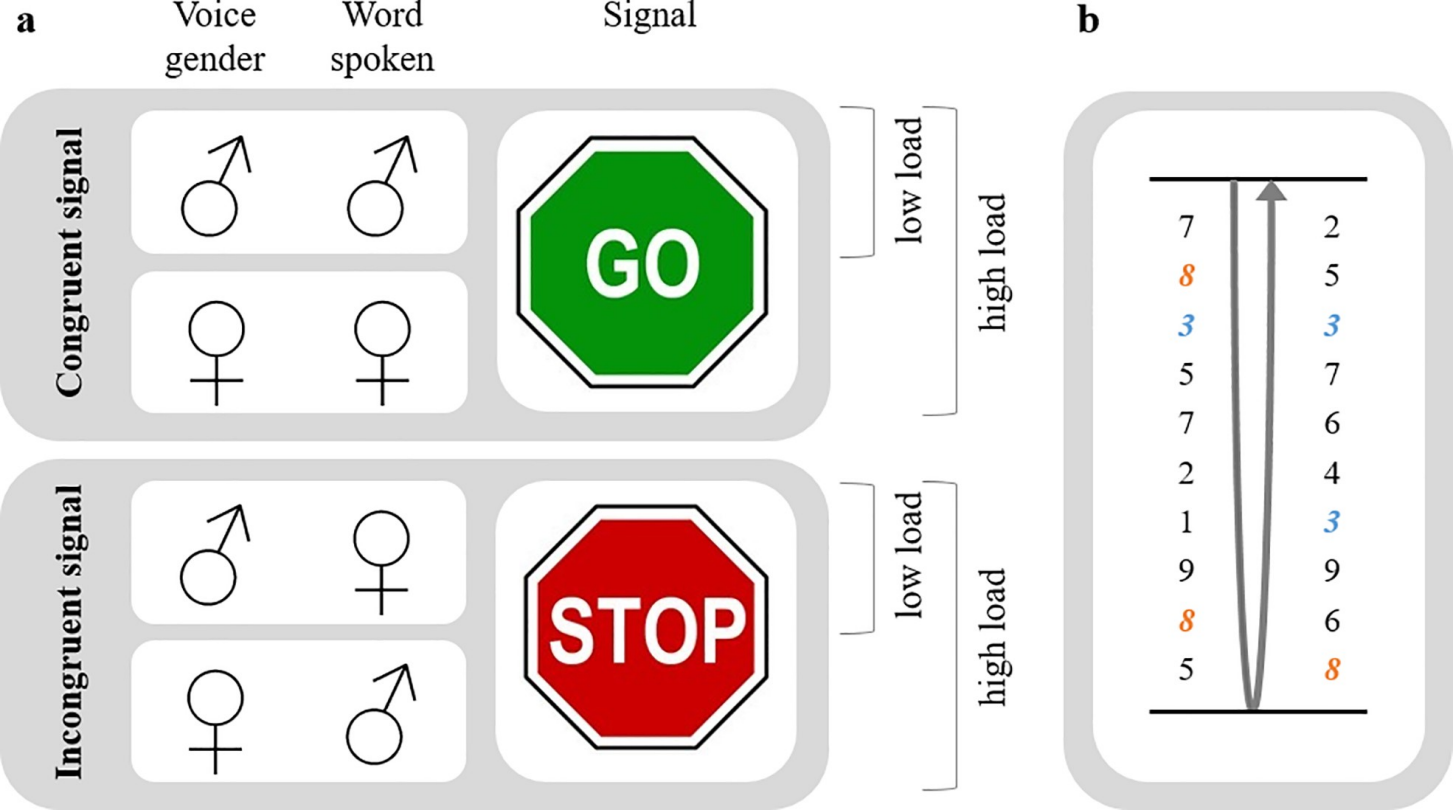
Cognitive load conditions. a. Auditory Stroop Task (AST) with the congruent signals (e.g. male voice speaking the Dutch translation of ‘man’) signaling the participant to start or continue walking, and incongruent signals (e.g. ‘woman’ spoken by a male voice) signaling to stand still. The high load AST (‘AST_high’) consisted of only the male voice, the low load AST (‘AST_low’) of both a male and female voice. b. Random Numbers Task (RNT) in which the numbers 1 to 9 were played in a random order and at random time intervals. Participants were instructed to mentally count how often two (‘RNT_high’) or one (‘RNT_low’) given number(s) occurred in the sequence (here ‘3’ and ‘8’, represented in orange and blue).

The walking task consisted of a 15 m walk through a corridor, a 180° turn, and walking back at a comfortable pace. Trials lasted 30 seconds and their start and end were signaled by recorded voice commands. Participants were informed that the distance traveled and whether or not they reached a turn were not of importance. The walking tasks were performed under five different cognitive load conditions (Fig 1): high and low cognitive load AST (‘AST_high’ and ‘AST_low’) and RNT (‘RNT_high’ and ‘RNT_low’), and no additional cognitive load (‘noCL’). In the AST_high, recorded male and female voices speaking the Dutch translations of the words ‘man’ and ‘woman’ were played through speakers. Congruent Stroop-cues, i.e. ‘man’ by a male voice, represented the cue to start or continue walking. Incongruent Stroop-cues, such as ‘woman’ spoken by a male voice, indicated to (continue to) stand still. Per trial were 3 or 4 Stroop-cues, of which at least 2 were incongruent, played with random timing and order. The AST_low was similar to the AST_high, apart from that it contained only the male and not the female voice. In the RNT, records of spoken numbers 1 up to 9 were played through speakers in a random order and at random time intervals. Participants were instructed to mentally count how often two (RNT_high) or one (RNT_low) given number(s) occurred in the sequence. This count was asked after the trial to verify adherence to the task, but no feedback on the performance was given. In the noCL, no cognitive load was added. Trials were performed in the presence (‘VC’) or absence (‘noVC’) of visual cues, consisting of white bars of 18mm x 18mm x 914mm, equally spaced on the floor at 40% of the participant’s height, rounded to the nearest 5cm [25]. An experiment was divided into two blocks (‘noVC’ and ‘VC’). Each cognitive load condition (‘AST_high’, ‘AST_low’, ‘RNT_high’, ‘RNT_low’, and ‘CC’) occurred twice per block. The order of the visual and cognitive conditions was counterbalanced across subjects.

### Experiment 2

#### Study population

We included 20 patients fulfilling the UK Brain Bank criteria for PD, and experiencing FOG minimally twice a day (defined as a score of 3 on question 2 of the New Freezing of Gait Questionnaire [NFOGQ] [26]). (Table 1). Exclusion criteria included a mini mental state examination (MMSE) score <24, executive dysfunction defined as a frontal assessment battery (FAB) score <13, comorbidities causing severe gait impairment, or an inability to walk 150 meters unaided. Patients were tested during the dopaminergic OFF-state, at least 12 hours after the last intake of dopaminergic medication. In addition, 18 age-matched healthy controls without impairments of gait, vision or hearing, who had not participated in the first experiment, were included.

#### Experimental procedure

Walking tasks were similar to those described at ‘Experiment 1’, with the exceptions that the corridor was 30m long, and that chairs were placed back-to-back 50 cm apart at 10m and 20m to create passages. The walking tasks were performed under the cognitive load conditions ‘AST_high’ and ‘noCL’, and the visual cueing conditions ‘VC’ and ‘noVC’ as described above. Experiments were divided into two blocks (‘VC’ and ‘noVC’), subdivided into two sessions (‘AST_high’ and ‘noCL’), with 6 trials per session. The order of the blocks and sessions was counterbalanced.

### Data acquisition and preprocessing

In both experiments, motion data were collected with the MVN Awinda motion capture system (Xsens, Enschede, the Netherlands)[27–30], consisting of 17 IMUs with 3D gyroscopes, accelerometers, and magnetometers (60 Hz sampling frequency, 30 ms latency) attached to the feet (2), lower legs (2), upper legs (2), pelvis (1), hands (2), forearms (2), upper arms (2), sternum (1), shoulders (2), and head (1). Data were transmitted wireless to a laptop with MVN studio 4.4 software installed. Raw accelerometer and gyroscope data, together with orientation and position data calculated by MVN studio, were exported to Matlab R2017b (Mathworks, Inc., Natick, MA, USA; statistics toolbox installed) for the offline calculation of gait parameters [31], and statistical analysis.

Raw data were organized within the Global reference frame [32]. The gait events ‘heel contact’ and ‘toe off’ [33] were detected. Noise, extreme outlier values (outside median plus and minus 3*IQR) due to technical reasons, turning movements [34], gait arrests following incongruent Stroop-cues, step hesitations (defined as interruptions of alternate stepping), and FOG episodes (as assessed by clinical video annotation) were removed. Data of trials in which calibration of the MVN Awinda motion capture system was qualitatively poor were corrected by applying a correction factor for walked distance based on rightly calibrated trials of the same participant. The representation of the relative position of the feet was sensitive to the quality of calibration, affecting step but not stride parameters. Therefore, stride length and stride time, but not step parameters, were analyzed.

In experiment 2, the number and duration of FOG were scored by two independent and experienced raters from video recordings with the sound switched off. Disagreements were discussed until consensus was reached.

#### Study parameters

Gait parameters calculated in both experiments were: stride length and time plus their coefficients of variation, gait velocity, and cadence. In the PD group, the number of FOG episodes (nrFOG) and the percent time frozen (PTF)[35] were measured. Gait parameters were contrasted for 1) the AST vs. the RNT (experiment 1), 2) the AST vs. the noCL in the conditions without and with visual cues (experiment 1 and 2), 3) the interaction of cognitive load and visual cues (experiment 1 and 2), and 4) the effects of high versus low cognitive load (experiment 1). For contrast 1–3 in the first experiment were high and low load versions of the AST and the RNT combined into ‘AST’ and ‘RNT’. FOG parameters were contrasted for the AST vs. the noCL in the conditions with and without visual cues (experiment 2).

#### Statistical analysis

A statistical level of α = 0.05 was applied. Groups were compared for age with a two-sample *t*-test, and for gender with a Fisher’s exact test. The effects of cognitive load, visual cues, and high versus low cognitive load were evaluated by the Sign test. The interaction of cognitive load and visual cues was analyzed with the two-way repeated measures ANOVA. The effects of participant class were assessed by the three-way mixed model ANOVA (within-subject factors: cognitive load condition [AST vs. noCL] and visual cueing condition [VC vs. noVC]; between-subjects factor: participant class [patient vs. control]). Outliers were defined as values outside the median plus and minus 1.5 times the interquartile range. In the presence of outliers, analyses were performed with and without the participants in whom outliers occurred. Data represented in tables and figures include participants with outlier values. Normality of data distribution was assessed with visual inspection of histograms, boxplots and Q-Q plots, and checked with the Shapiro-Wilk test. If data of a condition were missing due to technical issues, the mean value of the opposite VC condition (e.g. ‘AST/VC’ instead of ‘AST/noVC’) was taken and analyses were performed with and without the participant in which the missing condition occurred. Consensus on the number and duration of FOG episodes between the two raters was assessed by a Spearman’s rank order correlation.

## Results

In experiment 1, data of one participant were discarded from the analyses because of technical issues. In one participant in experiment 1, data were missing for one condition (RNT_low, no visual cues). Imputation of data from the opposite cueing condition (RNT_low, with visual cues), or exclusion of this participant from the analysis resulted in similar results. Analyses were pursued including this participant, with imputed data.

### Effects of the AST on gait parameters and FOG

#### AST vs. RNT in healthy controls

**Fig 2:**
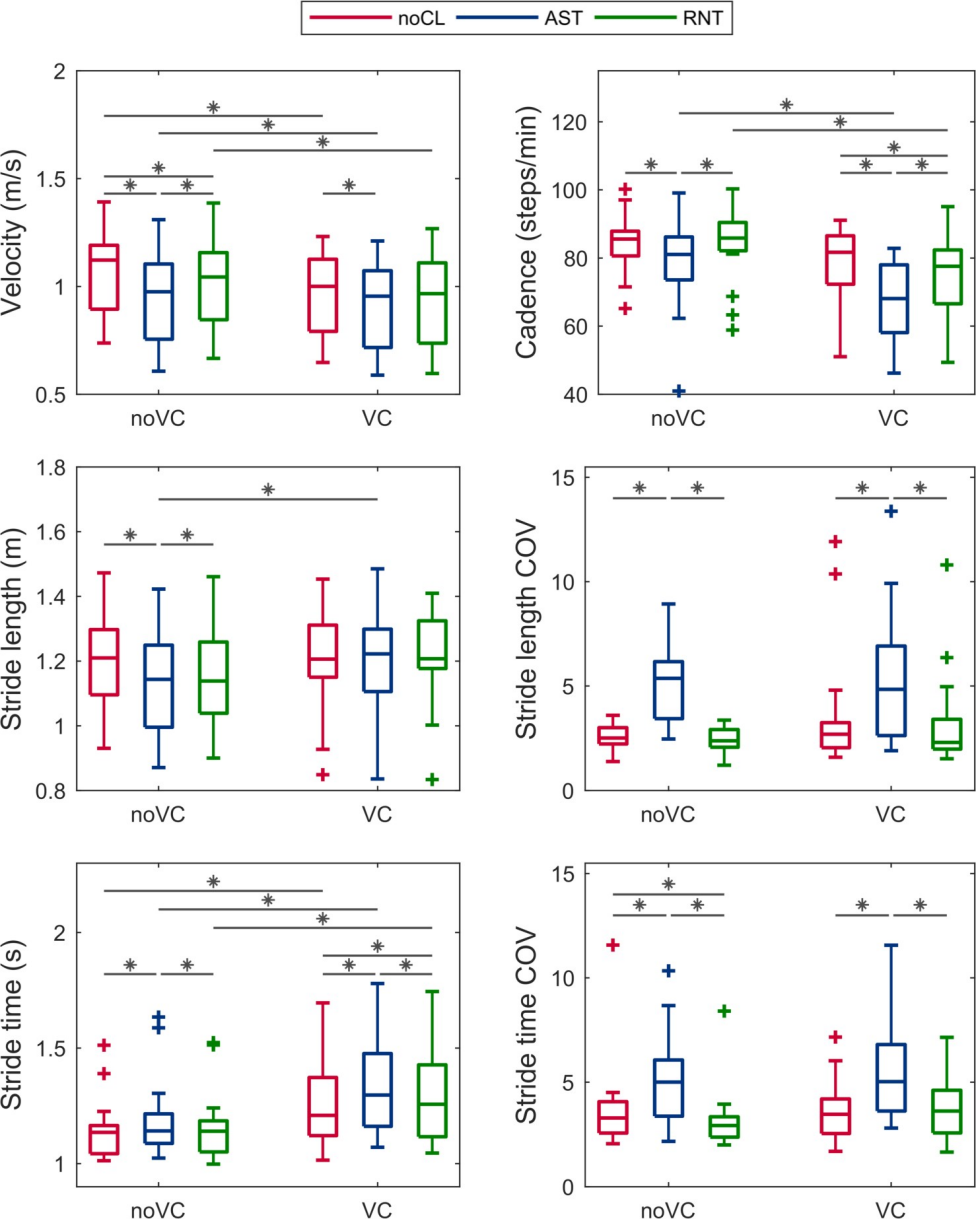
Gait parameters under different conditions of Experiment 1. Boxplots of the gait velocity (m/s), cadence (steps/min), stride length (m), stride length coefficient of variation (COV), stride time (sec), and stride time coefficient of variation (COV). Cognitive load conditions are illustrated in red (control cognitive load, noCL), blue (Auditory Stroop Task, AST), and green (Random Numbers Task, RNT). Conditions with no visual cues (‘noVC’) are displayed at the left sides of the plots, conditions with visual cues (‘VC’) at the right sides. Asterisks above indicate significant differences between cognitive load conditions (within the left or right half of a plot), and between visual cueing conditions (crossing the midline of the plot).

The AST resulted in a stronger reduction of velocity and cadence, and a stronger increase of the coefficients of variation of stride length and stride time (Table 2, Fig 2) than the RNT. Subsets of participants experienced lower stride length and higher stride time in the AST compared to the RNT in the absence of visual cues. However, because the majority of participants had similar stride lengths and stride times in both conditions, the differences between the medians of the conditions were equal or close to zero (Table 2).

**Table 2:** Results per aim/hypothesis.

Contrast	Exp	Group	Cues	Velocity (m/s)		Cadence (st/min)		Stride length (m)		Stride time (s)		Stride length COV		Stride time COV	
AST vs. RNT	1	HC	noVC	-0,07	**	-4,80	**	0,01	**	0,00	**	2,99	**	2,08	*
* *			VC	-0,01		-9,48	*	0,02		0,04	*	2,54	**	1,41	*
AST vs. noCL	1	HC	noVC	-0,15	**	-4,51	*	-0,07	**	0,01	**	2,86	**	1,72	**
		* *	VC	-0,05	*	-13,59	**	0,02		0,09	**	2,15	*	1,56	**
	2	HC	noVC	-0,08	**	-7,01	**	-0,07	**	0,00	*	6,51	**	3,76	**
			VC	-0,14	**	-13,8	**	-0,02		0,08	**	4,18	**	3,86	**
	2	PD	noVC	-0,19	**	-18,1	**	-0,07	**	0,08	**	13,60	**	6,12	**
			VC	-0,13	**	-17,2	**	-0,08	*	0,16	**	10,22	**	5,64	**
	2	PD vs. HC (F-ratio)	noVC & VC	1,787		0,472		6,510	*	3,358		2,671		1,042	
Interaction AST x cues (F-ratio)	1	HC		8,591	**	4,890	*	27,866	**	4,240		2,388		0,365	
	2	HC		0,121		3,835		8,648	**	7,486	*	4,697	*	0,705	
	2	PD		4,018		0,037		8,875	**	0,443		0,742		0,197	
	2	PD vs. HC		3,182		1,583		1,818		0,590		0,226		0,007	
High vs. low AST	1	HC	noVC	0,01		0,08		-0,01		0,00		3,16		0,16	
			VC	-0,06		-4,90		-0,01		0,00		0,58		-0,40	

Differences between the median values of conditions, unless stated otherwise. Asterisks indicate statistical significance

* p<0.05

** p<0.01.

AST, Auditory Stroop Task (high and low load, unless stated otherwise); COV, coefficient of variation; Exp, experiment; high, high cognitive load; low, low cognitive load; noCL, no additional cognitive load (control); noVC, no visual cues; RNT, Random Numbers Task (high and low load, unless stated otherwise); VC, with visual cues

#### Effects of the AST on gait parameters in PD patients and healthy controls

**Fig 3:**
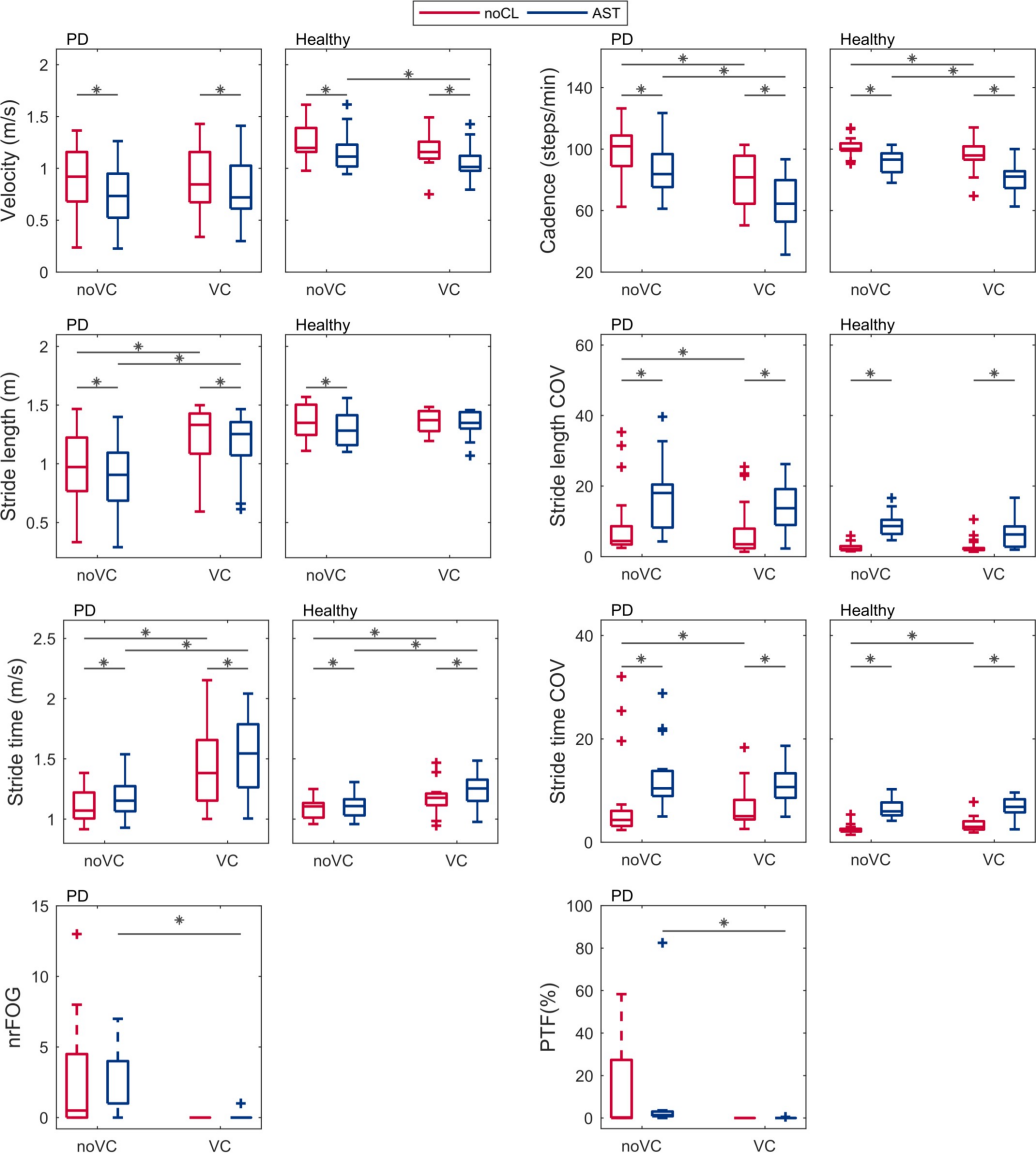
Gait parameters in different conditions of Experiment 2. Boxplots of the gait velocity (m/s), cadence (steps/min), stride length (m), stride length coefficient of variation (COV), stride time (sec), and stride time coefficient of variation (COV) for persons with Parkinson’s disease and freezing of gait (‘PD’) and healthy control subjects (‘Healthy’). The number of freezing of gait episodes (nrFOG) and percent time frozen (PTF) are given for PD patients. Cognitive load conditions are illustrated in red (control cognitive load, noCL), and blue (Auditory Stroop Task, AST). Conditions with no visual cues (‘noVC’) are displayed at the left sides of the plots, conditions with visual cues (‘VC’) at the right sides. Asterisks above indicate significant differences between cognitive load conditions (within the left or right half of a plot), and between visual cueing conditions (crossing the midline of the plot).

In the absence of visual cues and compared to the condition with no additional cognitive load, the AST reduced velocity, cadence and stride length, and increased the coefficients of variation of stride time and stride length in both PD patients and healthy controls (Table 2, Figs 2 and 3). The AST increased stride time in PD patients and in a subset of healthy controls, whilst the majority of healthy controls had similar stride times in both conditions (causing a difference between the medians close or equal to zero) (Table 2).

#### Effects of the AST on FOG occurrence in PD patients

The degree of consensus on rating the number [rs(18) = 0.657, p = 0.001] and duration of FOG [rs(18) = 0.668, p = 0.001] between raters was high. Eight PD patients experienced FOG at least once in the experiment, with in total 43 FOG episodes (median 1.5, first quartile 1, third quartile 8.5), which lasted 371.5 seconds (median 3.5 sec, first quartile 2.5 sec, third quartile 52.5 sec) in total. One participant accounted for 46.5% of all FOG episodes and was frozen for 35.2% of the time, and was considered an extreme outlier.

Compared to the condition with no additional cognitive load, the AST did not increase the number of FOG episodes (difference between medians 0.5, p-value 1.00) nor the percent time frozen (difference between medians 1.11, p-value 0.73) in the absence of visual cues, regardless of whether the extreme outlier was excluded. In the presence of visual cues, FOG occurred twice in one participant in the AST condition, and never in the cognitive control condition (difference between medians 0.0, p-value 1.00).

### Interaction between the AST and visual cues

Outlier values occurred in a minority of trials across study parameters and participants. If results changed significantly upon exclusion of participants in whom outlier values occurred, this has been described accordingly.

Both visual cues and the AST reduced velocity in the controls in the first experiment, with an only modest additional decrease due to the AST in the presence of visual cues (Table 2, Fig 2, S1 Table). However, these findings were not reproduced in both controls and patients in the second experiment (Table 2, Fig 3, S2 Table). The reduction in cadence caused by the AST was enforced in the presence of visual cues in both patients and controls, although statistical significance was only reached in the controls in the first experiment. The AST reduced stride length in the absence of visual cues (Table 2, Figs 2 and 3, S1 and S2 Tables). Visual cues centered stride length around twice the set distance between the cues. This was unchanged by the AST in controls, whilst in patients the AST caused a modest additional reduction in stride length in the presence of visual cues (S1 and S2 Tables, Figs 2 and 3). However, if participants with outlier values for stride length (N = 1) were removed from the analyses, significance of the interaction effect was lost in controls in the second experiment. There was a trend towards an increase in stride time due to the AST in the presence of visual cues. However, this was only statistically significant for controls in the second experiment. In the first experiment, statistical significance was reached only if participants with outlier values for stride time (N = 2) were excluded from the analysis (p = 0.004 without outliers; p = 0.054 including outliers). Considering the influence of outliers, and the inconsistent results between the two experiments, we warrant caution in interpreting this result. Only significant in controls in the second experiment, visual cues reduced the increase of the coefficient of variation of stride length caused by the AST. However, when participants with outlier values were excluded (N = 6), statistical significance for the interaction effect was lost. No interaction effects between the AST and visual cues existed for the coefficient of variation of stride time in both patients and controls. No statistically significant effects of participant class (controls and patients) were found on the interaction between the AST and visual cues on any of the gait parameters.

### Effects of high vs. low cognitive load AST

None of the gait parameters differed statistically significant between the high and low load AST in controls (Table 2, Fig 2, S1 Table).

## Discussion

This study primarily investigated whether the AST could be used to alter gait parameters in healthy elderly and in PD patients who experience FOG, and FOG parameters in PD patients, by increasing cognitive load. The AST caused both controls and patients to walk slower, with shorter and more variable steps. These results are consistent with prior studies reporting lower gait speed, shorter stride length, lower cadence and increased stride time and stride time variability under dual task conditions in controls [4], and PD patients [5]. The AST impacted gait parameters more than the RNT did, suggesting that the AST exerted the highest cognitive load. Alternatively, the additional motor task (i.e. gait cessation and initiation) present in the AST, but not the RNT, might compete for the same processing resources that gait control and the cognitive task call upon, leading to competition for limited resources and hence a deterioration in performance of (one of) the tasks, here walking [5]. These findings support our hypothesis that the AST influences gait in healthy elderly and PD patients. However, the AST did not increase the likelihood of FOG occurrence.

The secondary aim was to assess whether visual cues interfered with the influence of the AST on gait parameters in controls and PD patients. An interaction effect between cognitive load and visual cues was strongest and most consistent for stride length in both patients and controls. Both visual cues and cognitive load influenced stride length, but when applied simultaneously there was no additional effect of the AST on stride length in controls, and a modest additional reduction of the AST in patients. In healthy controls, interaction effects were additionally found for velocity, cadence, stride time, and stride length coefficient of variation, but the results were inconsistent between the two experiments and require replication in larger participant groups to assess their validity. A previous study found significant interactions between a dual task and visual cues for step length, step time, velocity and double support time percentage in persons with PD and FOG, indicating that visual cues prevented these gait parameters to be affected by dual tasks [10]. Our hypothesis that visual cues reduce the influence of cognitive load on gait and FOG was true for stride length, but not for the other gait parameters and FOG.

Our tertiary aim was to assess whether the size of the cognitive load exerted by the AST could be manipulated by different versions of the task. The high versus low load AST impacted gait parameters similarly, indicating that similar cognitive loads had been exerted. Neither version of the AST increased FOG severity in patients. Further work remains needed to identify which type of secondary task is better able to increase FOG occurrence and severity in gait experiments.

The main limitation of this study was the low number of participants, reducing the statistical power. Furthermore, patients and controls were not matched for gender. Although a previous study involving a gender-based auditory Stroop task found no significant differences in performance between male and female participants [36], we cannot exclude gender to have influenced differences found between patients and controls. In addition, a more thorough examination of cognitive function in both patients and controls would have allowed for a more detailed characterization of the participants.

We recommend future studies involving the AST to include more participants, and to match not only for age but also for gender and cognitive functioning.

## Conclusions

In conclusion, this study shows that the AST is well-suited to affect gait parameters by increasing cognitive load in gait experiments in healthy elderly and PD patients experiencing FOG. The AST did not affect FOG severity in PD patients. An interaction effect between cognitive load and visual cues was found for stride length. Interaction effects for velocity, cadence, stride time, and stride length coefficient of variation were inconsistent in our study and require replication to assess their validity. Nevertheless, interaction effects should be considered in studies investigating cognitive load and visual cues simultaneously.

## Supporting information

S1 Table Medians, interquartile ranges, and p-values of gait parameters in Experiment 1. Numbers indicate median (interquartile range), and p-values. COV, Coefficient of variation; AST, Auditory Stroop Task; RNT, Random Numbers Task; noCL, no additional cognitive load condition; VC, with visual cues; noVC, without visual cues.
(XLSX)

S2 Table Medians, interquartile ranges, and p-values of gait and freezing of gait parameters in Experiment 2. Numbers indicate median (interquartile range), and p-value. COV, Coefficient of variation; AST, Auditory Stroop Task; RNT, Random Numbers Task; noCL, no additional cognitive load condition; VC, with visual cues; noVC, without visual cues; Healthy, healthy control subjects; PD, persons with Parkinson's disease; PD (EO excl): extreme outlier (PD19) excluded from analysis; N/A, not applicable.
(XLSX)

## Abbreviations

AST: auditory Stroop task
AST_high: high load version of the auditory Stroop task
AST_low: low load version of the auditory Stroop task
FAB: Frontal Assessment Battery
FOG: freezing of gait
MMSE: mini-mental state examination
N-FOGQ: New Freezing of Gait Questionnaire
PD: Parkinson’s disease
RNT: random numbers task
RNT_high: high load version of the random numbers task
RNT_low: low load version of the random numbers task
UPDRS-part III: Unified Parkinson’s Disease Rating Scale part III (motor examination)
UPDRS-PIGD: Unified Parkinson’s Disease Rating Scale (postural instability and gait disorder)

## References

[1] Virmani T, Gupta H, Shah J, Larson-Prior L. Objective measures of gait and balance in healthy non-falling adults as a function of age. Gait & posture. 2018;65:100–5. 30558914 10.1016/j.gaitpost.2018.07.167 PMC9115806

[2] Woollacott M, Shumway-Cook A. Attention and the control of posture and gait: a review of an emerging area of research. Gait & posture. 2002;16(1):1–14. 12127181 10.1016/s0966-6362(01)00156-4

[3] Mazaheri M, Hoogkamer W, Potocanac Z, Verschueren S, Roerdink M, Beek PJ, et al Effects of aging and dual tasking on step adjustments to perturbations in visually cued walking. Experimental brain research. 2015;233(12):3467–74. 10.1007/s00221-015-4407-5 26298043 PMC4646946

[4] Al-Yahya E, Dawes H, Smith L, Dennis A, Howells K, Cockburn J. Cognitive motor interference while walking: a systematic review and meta-analysis. Neurosci Biobehav Rev. 2011;35(3):715–28. 10.1016/j.neubiorev.2010.08.008 20833198

[5] Kelly VE, Eusterbrock AJ, Shumway-Cook A. A review of dual-task walking deficits in people with Parkinson's disease: motor and cognitive contributions, mechanisms, and clinical implications. Parkinson's disease. 2012;2012:918719 10.1155/2012/918719 22135764 PMC3205740

[6] Bloem BR, Grimbergen YA, van Dijk JG, Munneke M. The "posture second" strategy: a review of wrong priorities in Parkinson's disease. Journal of the neurological sciences. 2006;248(1–2):196–204. 10.1016/j.jns.2006.05.010 16806270

[7] de Souza Fortaleza AC, Mancini M, Carlson-Kuhta P, King LA, Nutt JG, Chagas EF, et al Dual task interference on postural sway, postural transitions and gait in people with Parkinson's disease and freezing of gait. Gait & posture. 2017;56:76–81. 28521148 10.1016/j.gaitpost.2017.05.006 PMC5714292

[8] Bekkers EMJ, Dockx K, Devan S, Van Rossom S, Verschueren SMP, Bloem BR, et al The Impact of Dual-Tasking on Postural Stability in People With Parkinson's Disease With and Without Freezing of Gait. Neurorehabilitation and neural repair. 2018;32(2):166–74. 10.1177/1545968318761121 29554851

[9] Nutt JG, Bloem BR, Giladi N, Hallett M, Horak FB, Nieuwboer A. Freezing of gait: moving forward on a mysterious clinical phenomenon. The Lancet Neurology. 2011;10(8):734–44. 10.1016/S1474-4422(11)70143-0 21777828 PMC7293393

[10] Beck EN, Martens KAE, Almeida QJ. Freezing of Gait in Parkinson's Disease: An Overload Problem? PloS one. 2015;10(12). 10.1371/journal.pone.0144986 PMC4682987 26678262

[11] Ginis P, Nackaerts E, Nieuwboer A, Heremans E. Cueing for people with Parkinson's disease with freezing of gait: A narrative review of the state-of-the-art and novel perspectives. Ann Phys Rehabil Med. 2018;61(6):407–13. 10.1016/j.rehab.2017.08.002 28890341

[12] Ekker MS, Janssen S, Nonnekes J, Bloem BR, de Vries NM. Neurorehabilitation for Parkinson's disease: Future perspectives for behavioural adaptation. Parkinsonism & related disorders. 2016;22 Suppl 1:S73–7. 26362955 10.1016/j.parkreldis.2015.08.031

[13] Nieuwboer A, Kwakkel G, Rochester L, Jones D, van Wegen E, Willems AM, et al Cueing training in the home improves gait-related mobility in Parkinson's disease: the RESCUE trial. Journal of neurology, neurosurgery, and psychiatry. 2007;78(2):134–40. 10.1136/jnnp.200X.097923 17229744 PMC2077658

[14] Mancini M, Smulders K, Harker G, Stuart S, Nutt JG. Assessment of the ability of open- and closed-loop cueing to improve turning and freezing in people with Parkinson's disease. Sci Rep. 2018;8(1):12773 10.1038/s41598-018-31156-4 30143726 PMC6109152

[15] Nanhoe-Mahabier W, Delval A, Snijders AH, Weerdesteyn V, Overeem S, Bloem BR. The possible price of auditory cueing: influence on obstacle avoidance in Parkinson's disease. Movement disorders: official journal of the Movement Disorder Society. 2012;27(4):574–8. 22344625 10.1002/mds.24935

[16] Baker K, Rochester L, Nieuwboer A. The immediate effect of attentional, auditory, and a combined cue strategy on gait during single and dual tasks in Parkinson's disease. Archives of physical medicine and rehabilitation. 2007;88(12):1593–600. 10.1016/j.apmr.2007.07.026 18047873

[17] Baker K, Rochester L, Nieuwboer A. The effect of cues on gait variability—reducing the attentional cost of walking in people with Parkinson's disease. Parkinsonism & related disorders. 2008;14(4):314–20. 17988925 10.1016/j.parkreldis.2007.09.008

[18] Lohnes CA, Earhart GM. The impact of attentional, auditory, and combined cues on walking during single and cognitive dual tasks in Parkinson disease. Gait & posture. 2011;33(3):478–83. 21273075 10.1016/j.gaitpost.2010.12.029

[19] Rochester L, Nieuwboer A, Baker K, Hetherington V, Willems AM, Chavret F, et al The attentional cost of external rhythmical cues and their impact on gait in Parkinson's disease: effect of cue modality and task complexity. Journal of neural transmission (Vienna, Austria: 1996). 2007;114(10):1243–8. 10.1007/s00702-007-0756-y 17598068

[20] Amboni M, Cozzolino A, Longo K, Picillo M, Barone P. Freezing of gait and executive functions in patients with Parkinson's disease. Movement disorders: official journal of the Movement Disorder Society. 2008;23(3):395–400. 18067193 10.1002/mds.21850

[21] Strouwen C, Molenaar EA, Keus SH, Munks L, Heremans E, Vandenberghe W, et al Are factors related to dual-task performance in people with Parkinson's disease dependent on the type of dual task? Parkinsonism & related disorders. 2016;23:23–30. 26683745 10.1016/j.parkreldis.2015.11.020

[22] Knight S, Heinrich A. Different Measures of Auditory and Visual Stroop Interference and Their Relationship to Speech Intelligibility in Noise. Front Psychol. 2017;8:230 10.3389/fpsyg.2017.00230 28367129 PMC5355492

[23] Shor RE. An auditory analog of the Stroop Test. J Gen Psychol. 1975;93(2d Half):281–8. 1194907

[24] Green EJ, Barber PJ. An auditory Stroop effect with judgements of speaker gender. Percept Psychophys. 1981;30(5):459–66. 7329763 10.3758/bf03204842

[25] Janssen S, Bolte B, Nonnekes J, Bittner M, Bloem BR, Heida T, et al Usability of Three-dimensional Augmented Visual Cues Delivered by Smart Glasses on (Freezing of) Gait in Parkinson's Disease. Frontiers in neurology. 2017;8:279 10.3389/fneur.2017.00279 28659862 PMC5468397

[26] Nieuwboer A, Rochester L, Herman T, Vandenberghe W, Emil GE, Thomaes T, et al Reliability of the new freezing of gait questionnaire: agreement between patients with Parkinson's disease and their carers. Gait & posture. 2009;30(4):459–63. 19660949 10.1016/j.gaitpost.2009.07.108

[27] XT B.V. Xsens MTw Awinda system [Available from: https://www.xsens.com/products/mtw-awinda/.

[28] Al-Amri M, Nicholas K, Button K, Sparkes V, Sheeran L, Davies JL. Inertial Measurement Units for Clinical Movement Analysis: Reliability and Concurrent Validity. Sensors (Basel, Switzerland). 2018;18(3). 10.3390/s18030719 PMC5876797 29495600

[29] Paulich M, Schepers M, Rudigkeit N, Bellusci G. Xsens MTw Awinda: Miniature wireless inertial-magnetic motion tracker for highly accurate 3D kinematic applications *(white paper)*: XSENS TECHNOLOGIES B.V.; 2018 [Available from: https://www.xsens.com/download/pdf/MTwAwinda_WhitePaper.pdf.

[30] Roetenberg D, Luinge H, Slycke P. Xsens MVN: full 6DOF human motion tracking using miniature inertial sensors: Technical Report. 2009.

[31] Zhao Y, Nonnekes J, Storcken EJ, Janssen S, van Wegen EE, Bloem BR, et al Feasibility of external rhythmic cueing with the Google Glass for improving gait in people with Parkinson's disease. Journal of neurology. 2016;263(6):1156–65. 10.1007/s00415-016-8115-2 27113598 PMC4893372

[32] Karatsidis A, Bellusci G, Schepers HM, de Zee M, Andersen MS, Veltink PH. Estimation of Ground Reaction Forces and Moments During Gait Using Only Inertial Motion Capture. Sensors (Basel, Switzerland). 2016;17(1). 10.3390/s17010075 PMC5298648 28042857

[33] Skog I, Handel P, Nilsson JO, Rantakokko J. Zero-velocity detection—an algorithm evaluation. IEEE Trans Biomed Eng. 2010;57(11). 10.1109/TBME.2010.2060723 20667801

[34] Beyea J, McGibbon CA, Sexton A, Noble J, O'Connell C. Convergent Validity of a Wearable Sensor System for Measuring Sub-Task Performance during the Timed Up-and-Go Test. Sensors (Basel, Switzerland). 2017;17(4). 10.3390/s17040934 PMC5426930 28441748

[35] Morris TR, Cho C, Dilda V, Shine JM, Naismith SL, Lewis SJ, et al A comparison of clinical and objective measures of freezing of gait in Parkinson's disease. Parkinsonism & related disorders. 2012;18(5):572–7. 22445248 10.1016/j.parkreldis.2012.03.001

[36] Christensen TA, Lockwood JL, Almryde KR, Plante E. Neural substrates of attentive listening assessed with a novel auditory Stroop task. Frontiers in human neuroscience. 2011;4:236 10.3389/fnhum.2010.00236 21258643 PMC3020403

